# Normal-appearing brain tissue analysis in radiologically isolated syndrome using 3 T MRI

**DOI:** 10.1097/MD.0000000000004101

**Published:** 2016-07-08

**Authors:** Andrés Labiano-Fontcuberta, Virginia Mato-Abad, Juan Álvarez-Linera, Juan Antonio Hernández-Tamames, María Luisa Martínez-Ginés, Yolanda Aladro, Lucía Ayuso, Ángela Domingo-Santos, Julián Benito-León

**Affiliations:** a Department of Neurology, University Hospital “12 de Octubre” Madrid; b Neuroimaging Laboratory, Center for Biomedical Technology, Rey Juan Carlos University, Móstoles; c Department of Radiology, Hospital Ruber International, Madrid; d Department of Neurology, University Hospital “Gregorio Marañón,” Madrid; e Department of Neurology, University Hospital of Getafe, Getafe; f Department of Neurology, University Hospital “Principe de Asturias” Alcalá de Henares; g Centro de Investigación Biomédica en Red sobre Enfermedades Neurodegenerativas (CIBERNED); h Department of Medicine, Complutense University, Madrid, Spain.

**Keywords:** case–control study, magnetic resonance imaging, pathophysiology, proton magnetic resonance spectroscopy, radiologically isolated syndrome

## Abstract

To date, it remains largely unknown whether there is in radiologically isolated syndrome (RIS) brain damage beyond visible T2 white matter lesions. We used single- voxel proton magnetic resonance spectroscopy and diffusion tensor imaging (3 T MRI) to analyze normal-appearing brain tissue regions in 18 RIS patients and 18 matched healthy controls. T2-hyperintense lesion volumes and structural brain volumes were also measured. The absolute metabolite concentrations and ratios of total N-acetylaspartate+N-acetylaspartyl glutamate (NAA), choline-containing compounds, myoinositol, and glutamine-glutamate complex to creatine were calculated. Spectral analysis was performed by LCModel. Voxelwise morphometry analysis was performed to localize regions of brain tissue showing significant changes of fractional anisotropy or mean diffusivity. Compared with healthy controls, RIS patients did not show any significant differences in either the absolute concentration of NAA or NAA/Cr ratio in mid-parietal gray matter. A trend toward lower NAA concentrations (–3.35%) was observed among RIS patients with high risk for conversion to multiple sclerosis. No differences in the other metabolites or their ratios were observed. RIS patients showed lower fractional anisotropy only in clusters overlapping lesional areas, namely in the cingulate gyrus bilaterally and the frontal lobe subgyral bilaterally (*P* < 0.001). Normalized brain and cortical volumes were significantly lower in RIS patients than in controls (*P* = 0.01 and *P* = 0.03, respectively). Our results suggest that in RIS, global brain and cortical atrophy are not primarily driven by significant occult microstructural normal appearing brain damage. Longitudinal MRI studies are needed to better understand the pathological processes underlying this novel entity.

## 1 Introduction

The steady increase in the use of magnetic resonance imaging (MRI) for the evaluation of medical conditions, such as headaches or dizziness, has led to the emergence of a new condition named radiologically isolated syndrome (RIS), which is characterized by incidental brain MRI finding of white matter lesions demonstrating dissemination in space in subjects with a normal neurologic examination, and without historical accounts of typical multiple sclerosis (MS) symptoms.^[1]^

There is a growing amount of evidence suggesting that RIS and MS patients share both nonmotor clinical features^[2–5]^ and quantitative brain tissue damage.^[6–12]^ Accordingly, approximately one-third of RIS subjects, especially those with spinal cord involvement, will present a future demyelinating event within 5 years after diagnosis.^[13]^ However, to date it remains largely unknown whether there is in RIS brain damage beyond visible T2 white matter lesions. There is therefore a need to carry out quantitative MRI-based techniques to gain a better insight into the pathophysiology nature of this complex entity. In this context, several quantitative MRI methods have been established to assess changes in brain areas, which appear to be normal on conventional MRI, the so-called normal-appearing gray and white matter. Diffusion tensor imaging (DTI) has the potential to quantify microstructural changes that modify the integrity of brain tissues.^[14]^ By using DTI, the brain tissue microstructure can be determined by quantitative indexes, such as mean diffusivity, which is affected by cellular size and integrity, and fractional anisotropy, which reflects the degree of alignment of cellular structures within fiber tracts and their structural integrity.^[14]^ Additionally, proton magnetic resonance spectroscopy (1H-MRS), a noninvasive technique, is widely used to assess the neurometabolic profile of neurological diseases.^[15]^

The main metabolites detected by 1H-MRS are *N*-acetylaspartate+*N*-acetylaspartyl glutamate (hereinafter referred to as NAA), creatine (Cr), choline-containing compounds (Cho), glutamine-glutamate complex (hereinafter referred to as Glx), and myo-inositol (MI).^[15]^ NAA is mainly found within neurons, and a reduction of this metabolite reflects neuronal dysfunction or loss.^[15]^ Cr provides an energy buffer in the brain, and it includes Cr and phosphocreatine.^[15]^ As an internal reference, it is stable in different pathophysiological conditions.^[15]^ Cho is involved in both synthesis and breakdown of phospholipid membranes.^[15]^ Glx is an excitatory neurotransmitter and found in early neuronal damage.^[15]^ MI is a marker of glial cells and, therefore, its concentration is proportional with the extent of gliosis.^[15]^

The aim of the present study was to assess the presence or not of microstructural damage in normal-appearing brain regions of a cohort of RIS patients by combining 1H-MRS, DTI, and voxelwise morphometry analysis.

## 2 Methods

### 2.1 Study design and subjects

A total of 18 RIS patients, most of whom came from already existing MS databases, were recruited at 4 MS clinics in Madrid (Spain). These patients had come to our attention after undergoing conventional brain 1.5 T MRI for various medical conditions not suggestive of MS. Reasons for these first MRIs, which were performed a mean of 4.02 years earlier, were: headache (N = 5), dizziness (N = 5), tinnitus/ hypoacusia (N = 3), syncope (N = 1), restless legs syndrome (N = 1), research control (N = 1), traffic accident (N = 1), and prolactinoma (N = 1).

Brain white matter abnormalities were initially identified by a neuroradiologist and subsequently examined by an MS specialist at each clinical site to guarantee they fulfilled the Okuda criteria for RIS,^[1]^ which imply (1) the presence of white matter abnormalities suggestive of a demyelinating process (ovoid, well-circumscribed and measuring >3 mm^2^) that satisfied Barkhof criteria (at least 3 of 4 criteria) for dissemination in space;^[16,17]^ (2) not better accounted for by other disease processes, such as, in particular, vascular disease; and (3) no apparent impact on everyday functioning.

After the initial MRI imaging, all patients underwent cervical spinal MRI but only a subgroup (50%) agreed to undergo lumbar puncture for cerebrospinal analysis. An extensive neurologic examination and an accurate clinical history were performed by a neurologist to rule out both any neurologic sign and history of remitting clinical symptoms lasting >24 hours consistent with MS. In addition, they underwent a complete nonstandardized workup for the evaluation of other medical conditions that could explain the observed lesions on brain MRI.

A control group consisting of 18 healthy volunteers (13 women, 5 men; mean age 41 years, range 27–56 years) with no history of known psychiatric or neurological disorders, was recruited either from relatives or friends from health professionals at the University Hospital “12 de Octubre” of Madrid (Spain).

Once the study was described to subjects and written (signed) informed consent was obtained from all enrollees, a combined 1H-MRS spectroscopy and multisequence MRI examinations were acquired in a single session using a single 3 T scanner at CIEN (Center for Research on Neurological diseases, in Spanish) Foundation in Madrid (Spain). Psychiatric and neuropsychological tests were conducted in a single session by experienced clinical neuropsychologists who were blinded to the clinical status during an interview during the week in which they had completed the aforementioned MRI examination.

All procedures were approved by the ethical standards committees on human experimentation at the University Hospital “12 de Octubre” (Madrid).

### 2.2 Measurement instruments

Cognitive functioning was performed through the Rao Brief Repeatable Battery.^[18]^ The Stroop test was administered to evaluate executive functions.^[19]^ Depression severity was measured by the original 17-item version of the Hamilton Depression Rating Scale.^[20]^

### 2.3 MRI acquisition

All MRI data were acquired with a clinical 3 T Signa HDx MR scanner (GE Healthcare, Waukesha, WI) using an 8-channel phased array coil. The imaging protocol included a 3D T1-weighted SPGR with a TR = 10.012 ms, TE = 4.552 ms, TI = 600 ms, NEX = 1, acquisition matrix = 288 × 288, full brain coverage, resolution = 0.4688 × 0.4688 × 1 mm, flip angle = 12.

**Figure 1:**
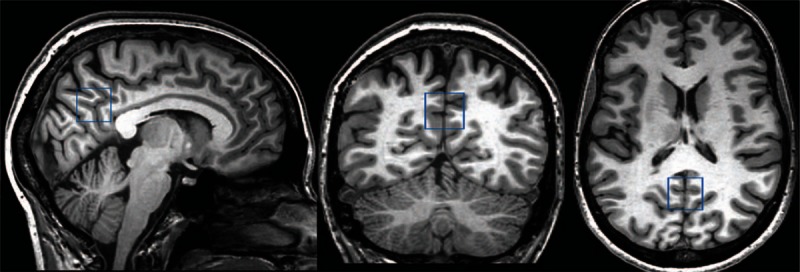
T1-weighted magnetic resonance images showing the region of interest location used for proton magnetic resonance spectroscopy of the mid-parietal gray matter.

The spectroscopic (1H-MRS) protocol consisted of a Point Resolved Spin Echo (PRESS) acquisition with TR = 2000 ms and TE = 35 ms. A single-voxel with a size of 10 ± 0.19 cm^3^ was selected in the mid-parietal gray matter. This single-voxel size mainly contained gray matter (around 65%), with some white matter (around 20%) and cerebrospinal fluid (around 15%). An example of the voxel placement is given in Fig. 1.

The DTI protocol acquisition consisted of 3 images without diffusion gradients (*b* = 0 s/mm^2^) followed by 45 images measured with 45 directions (*b* = 1000 s/mm^2^) isotropically distributed in space. Additional parameters of the acquisition were: TE = 85.3 ms, TR = 10.100 ms, flip-angle = 90, slice thickness = 3 mm (no gap), resolution = 2.6042 × 2.6042 × 2.6 mm, FOV = 250 mm and axial acquisition.

All MRI/1H-MRS acquisitions and image postprocessing (see below) were performed by a neuroradiologist (JA-L) and a physicist (VM-A) who were blinded to the clinical diagnoses.

### 2.4 1H-MRS and DTI post-processing

#### 2.4.1 1H-MRS

Metabolite quantification was performed with the LCModel software (http://www.s-provencher.com) for the automatic quantitation of in vivo 1H-MRS spectra.^[21]^ The LCModel method automatically performs phase adjustments, frequency alignment, baseline subtraction, and eddy current correction. Relative metabolite concentrations (and their uncertainties) are estimated by fitting the spectrum to a linear combination of “basis spectra” of each individual metabolite, obtained from solutions acting as concentration references for the in vivo acquisitions. For this study, a basis set provided by the LCModel software for a 3 T PRESS acquisition with TE = 35 ms was used. The unsuppressed water spectrum is then used to normalize the initial fit to generate a first estimate of metabolite concentration in the tissue. LCModel defines the concentrations of the pertinent metabolites by scaling the relative areas and chemical shifts across the 2 sets of spectra. The fitting of the spectral peaks was thus achieved with *a priori* knowledge of their actual characteristics.

The main brain metabolites of interest were the NAA peak at 2.0 ppm, the Cr peak at 3.0 ppm, the Cho peak at 3.5 ppm, the MI peak at 3.5 ppm, and the Glx peak at 2.2–2.4 ppm.

To define a criterion for rejection of poor quality spectra, the Cramer Rao lower bounds (given as %Standard deviation [SD]-value by the LCModel) were used. Only those spectra with a %SD < 20 were included in the study. In all these spectra, the linewidth, obtained during the shimming process, was (on average) 6.45 with no significant mean differences between the groups.

Finally, metabolite concentrations obtained from the LCModel were corrected for gray and white matter and cerebrospinal fluid content.^[22]^ The 3D high-resolution T1-weighted images were segmented into gray matter, white matter, and cerebrospinal using SPM-8 toolbox (www.fil.ion.ucl.ac.uk/spm/) to determine the tissue composition of the voxel of interest. Metabolite quantification was adjusted for partial volume effects as previously described.^[22]^

#### 2.4.2 Diffusion-weighted imaging

Diffusion-weighted image data were pre-processed with FMRIB's Diffusion Toolbox (http://fsl.fmrib.ox.ac.uk/fsl/fslwiki/FslOverview/). Preprocessing consisted of Eddy current correction, motion correction, and the removal of non–brain tissue using the robust Brain Extraction Tool.^[23]^ DTI were created using the weighed least squares fitting method. We derived images of fractional anisotrophy and mean diffusivity from the DTI. To calculate the specific fractional anisotrophy and mean diffusivity values in the subcortical regions, we first obtained the subcortical volume measurements from the 3D T1 MRI images using the freely available software FreeSurfer (http://surfer.nmr.mgh.harvard.edu/). FreeSurfer is a popular and publicly available software package for studying cortical and subcortical anatomy. Briefly, images underwent preprocessing including intensity normalization and skull stripping, which was followed by labeling of cortical and subcortical regions. FreeSurfer's main cortical reconstruction pipeline begins with registration of the structural volume with the Talairach atlas. After bias field estimations and the removal of bias, the skull is stripped and subcortical white and gray matter structures are segmented.^[24]^ Then, the fractional anisotrophy and mean diffusivity maps were resampled by means of a rigid-body transformation from the diffusion space to the freesurfer's structural space. After that, the fractional anisotrophy and mean diffusivity values from the Freesurfer's subcortical regions were computed.

Also, a voxelwise morphometry analysis pipeline was used to find differences in fractional anisotrophy and mean diffusivity between groups. Voxelwise morphometry analysis of fractional anisotrophy and mean diffusivity images were carried out with the SPM8 software (www.fil.ion.ucl.ac.uk/spm/software/spm8/). First, b0 images were manually aligned to the anterior commissure–posterior commissure line, and the same alignment was applied to the fractional anisotrophy images. Then, these fractional anisotrophy images were coregistered to a fractional anisotrophy template from the FMRIB Software LIbrary (http://fsl.fmrib.ox.ac.uk/fsl/fslwiki/) using linear affine registration with normalized mutual information as the fitness function. The same spatial transformation was applied to the mean diffusivity maps. The registered images were normalized to the fractional anisotrophy template using a nonlinear registration algorithm^[25]^ and were then smoothed with a 3D Gaussian kernel (4-mm full wide half maximum [FWHM]). After smoothing, statistical analysis of the results was carried out using a 2-sample T test applied to each voxel in order to assess differences in the means.

#### 2.4.3 White matter lesion volume analysis

T2 hyperintense lesions were segmented in FLAIR images by employing the automated lesion growth algorithm^[26]^ as implemented in the LST toolbox version 2.0.11 (www.statisticalmodelling.de/lst.html) for Statistical Parametric Mapping. Lesion mask were estimated and then transformed into standard space and averaged to yield a mean lesion mask across subjects.

#### 2.4.4 Brain volume analysis

Normalized brain volumes were quantified on high-resolution T1-weighted image using the SIENAx method, part of the FMRIB Software Library.^[27]^

### 2.5 Statistical analysis

The data were conducted using the SPSS Version 21.0 (SPSS, IBM Corporation). A nominal *P* value < 0.05 was regarded as significant using the 2-tailed test. Using the Shapiro–Wilk test, we determined that age, metabolite concentrations, brain volumes, and DTI measures of cortical and subcortical structures were normally distributed (Shapiro–Wilk test, *P* > 0.05 for all of them), whereas white matter lesion volume was not normally distributed (Shapiro–Wilk test, *P* < 0.001).

Differences between the groups were then tested using 2 independent sample *t*-tests for continuous and normally distributed data and χ2 test or Fisher's exact test to analyze categorical variables. The relationships between metabolite measures and clinical parameters (cognitive and psychiatric measures) and brain volumes were assessed using Pearson's correlation. Correlations of 1H-MRS findings with white matter lesion volume were assessed using the nonparametric Spearman's rank correlation.

Differences in fractional anisotropy and mean diffusivity between groups were obtained by performing a voxelwise morphometry analysis by means of the *T*-test, with age and gender as covariates. The result of the statistical analysis was a *T* map that showed the differences between the control and patient group. Correction for multiple comparisons was applied to test these differences between groups. A threshold of *P* < 0.001 was considered statistically significant.

## 3 Results

### 3.1 Sample characteristics

Table 1 summarizes the demographic and clinical characteristics of RIS patients and healthy controls. Nine (50%) of the 18 RIS patients failed in at least 1 cognitive test, defined as a Z score ≤ 2.0 standard deviations (SD) below the healthy controls mean of any of the cognitive tests. The RIS group was stratified into RISK+ (higher risk for conversion to future MS) versus RISK– (lower risk for conversion to future MS) according to (a) the presence of lesions within the spinal cord or (b) no lesions of the spinal cord, but the presence of at least 2 of the following characteristics: abnormal cerebrospinal fluid, gadolinium enhancing lesions, or dissemination in time. Among the 6 RIS subjects who were classified as RISK+, 4 were included based on spinal cord lesions criteria, and 2 on the presence of several risk criteria for conversion to MS.

**Table 1:**
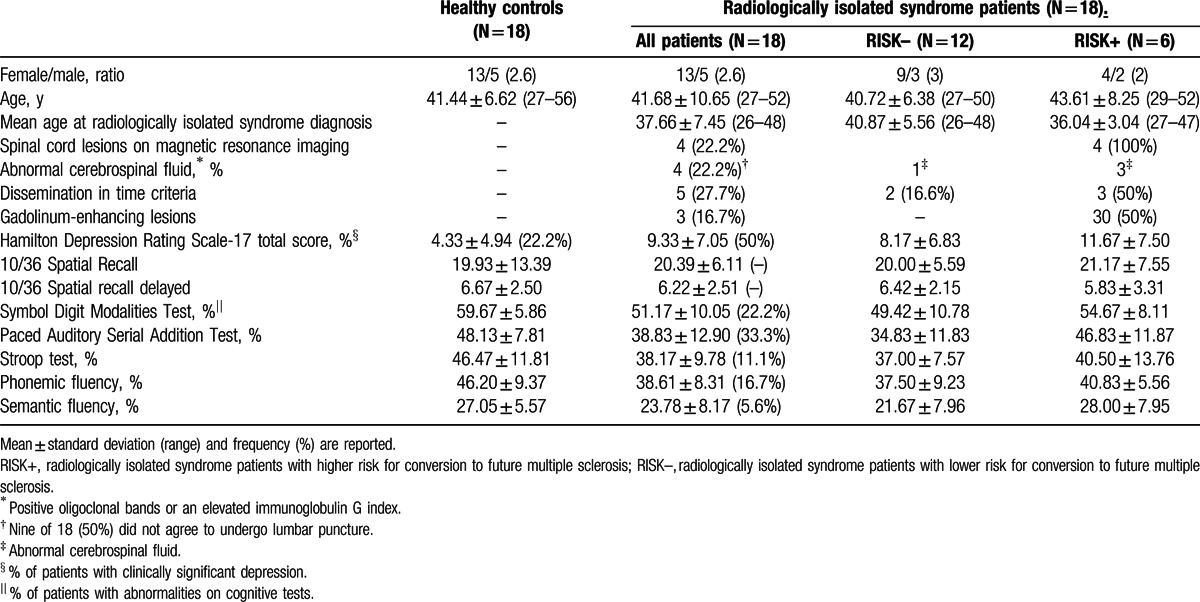
Demographic and clinical characteristics of the sample.

### 3.2 1H-MRS results

There was no statistically significant difference between the RIS patients and the healthy controls in either the absolute concentration of NAA (11.35 ± 0.65 vs 11.25 ± 0.56, *P* = 0.62) or NAA/Cr ratio (1.47 ± 0.94 vs 1.45 ± 0.83, *P* = 0.52). However, the RIS patients who were classified as RISK+ showed a trend toward a lower absolute concentration of NAA than both the RISK– subgroup (–3.69%) (*P* = 0.12) and healthy controls (–3.35%) (*P* = 0.15). No differences in the other metabolites or their ratios were observed (Table 2).

**Table 2:**
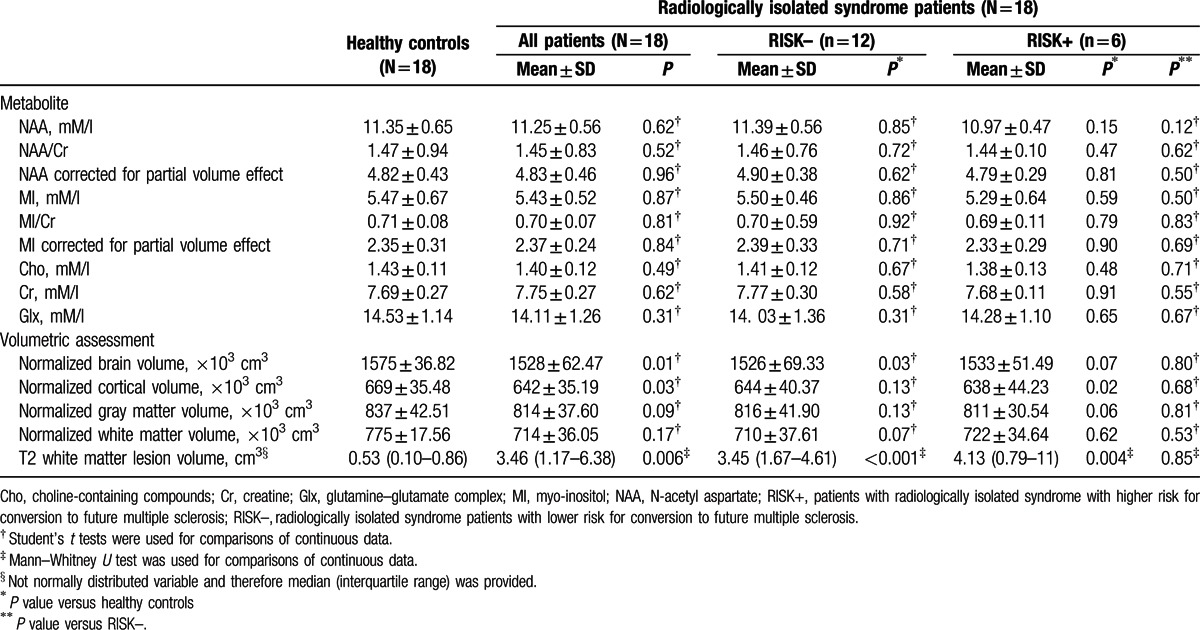
Magnetic resonance imaging characteristics of healthy controls and radiologically isolated syndrome patients.

No significant correlation was found between the absolute concentration of NAA and T2 white matter lesion volume (*r* = –0.34, *P* = 0.16). However, a slightly stronger inverse correlation was observed between the absolute concentration of NAA corrected for partial volume effect^[22]^ and T2 white matter lesion volume (*r* = –0.44, *P* = 0.08). Among all the correlations analyzed, the strongest, yet modest, were found between the absolute concentration of NAA corrected for the partial volume effect^[22]^ and the brain volume measures. Specifically, in RIS patients, the absolute concentration of NAA corrected for the partial volume effect^[22]^ was correlated with normalized brain volume (*r* = 0.54, *P* = 0.02), normalized white matter volume (*r* = 0.48, *P* = 0.04), normalized gray matter volume (*r* = 0.44, *P* = 0.06), and normalized cortical volume (*r* = 0.44, *P* = 0.07). No correlations were found between 1H-MRS findings and clinical parameters.

### 3.3 Diffusion tensor analysis

**Figure 2:**
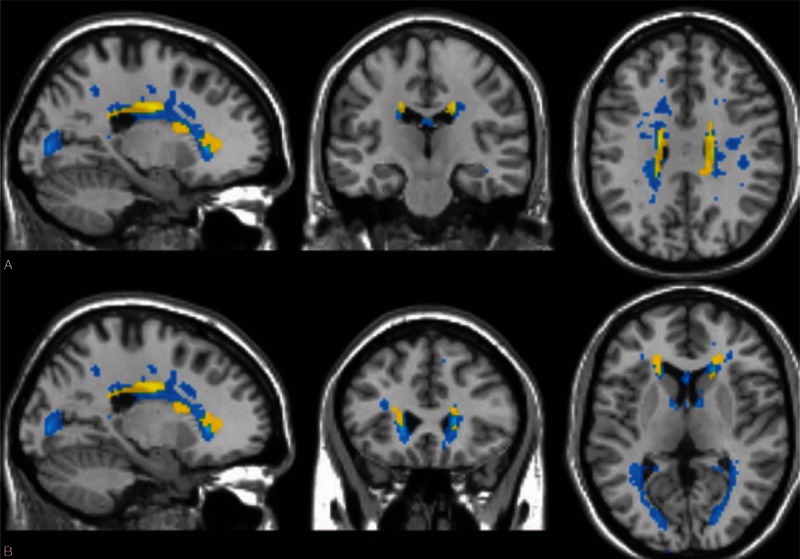
Voxelwise morphometry analysis results obtained using fractional anisotrophy maps and mean lesion mask. Saggital, coronal, and axial views are presented. Clusters of reduced fractional anisotrophy in RIS patients compared with healthy controls (*P* < 0.001 corrected for multiple comparisons) are shown in yellow and average lesion mask is shown in blue. The overlay of the significant map clusters on the mean lesion mask shows that most of the abnormalities highlighted by voxelwise morphometry analysis were primary located within lesions. (A) Cingulate gyrus. (B) Frontal lobe subgyral. RIS, radiologically isolated syndrome.

Figure 2 and Table 3 show the results of the voxelwise morphometry analysis method using the fractional anisotropy maps. Note that the clusters with altered microstructural integrity overlap with lesion mask and are dominant in the cingulate gyrus bilaterally (Fig. 2A) and in the frontal lobe subgyral bilaterally (Fig. 2B) (*P* < 0.001).

**Table 3:**
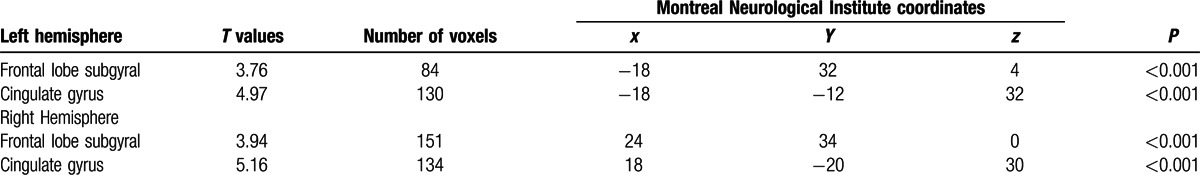
Voxelwise morphometry analysis of reduced fractional anisotropy in radiologically isolated syndrome patients compared with control group.

No fractional anisotropy or mean diffusivity differences were detected in the subcortical deep gray mater structures (thalamus, caudate, putamen, and hippocampus, amygdala) between RIS and healthy control groups (*P* > 0.10 for all).

### 3.4 Brain volume analysis

Normalized brain (1575 ± 36.82 vs 1528 ± 62.47 [cm^3^ × 103], *P* = 0.01) and normalized cortical (669 ± 35.48 vs 642 ± 35.19 [cm^3^ × 103], *P* = 0.03) volumes were significantly lower in the RIS group than in controls. T2 white matter lesion volume was correlated with both normalized cortical volume (*r* = –0.59, *P* = 0.012) and normalized gray matter volume (*r* = –0.52, *P* = 0.033). No significant brain volumes differences were found when RIS patients where subdivided according to the presence/absence of cognitive impairment. In terms of Spearman's correlations, no associations were found between psychiatric measures and white matter lesion or brain volumes.

## 4 Discussion

This study combines, to our knowledge for the first time, an holistic assessment of the brain damage occurring in normal-appearing brain regions of RIS patients using 1-HMRS and DTI analysis.

Compared to controls, the voxelwise morphometry analysis did not reveal diffuse fractional anisotropy or mean diffusivity changes in the principal white matter tracts of RIS patients. The results of the current research are in line with previous studies assessing microstructural integrity of white matter tracts in RIS subjects by using magnetization transfer ratio,^[6]^ and tract-based spatial statistics,^[9]^ further suggesting that occult microstructural normal-appearing white matter damage is not significantly present beyond T2 visible lesions. In addition, the present study extends these findings by reporting no DTI abnormalities in both regional cortical and subcortical deep gray matter structures by employing Freesurfer's segmentation. These results differ widely from a number of DTI studies, which identified a diffuse pattern of white matter microstructural abnormalities in the majority of white matter tracts and normal-appearing white matter in clinically isolated syndrome (CIS) patients suggestive of MS^[28–31]^ and benign MS patients.^[29,32]^ Taken together, these results suggest that RIS patients might have experienced more efficient reparative mechanisms after white matter injury, thereby contributing to explain, at least in part, the lack of clinical neurologic events suggestive of MS.

Given that DTI characteristics are not ideally suited to characterize gray matter of the brain due to its relatively isotropic structure, the MRI study approach was completed by using single-voxel 1-H-MRS located mainly on gray matter parietal lobe. Only 1 previous study has investigated metabolic changes in RIS subjects.^[8]^ In that study, Stromillo et al^[8]^ found a statistically significant decrease of the NAA/Cr ratio in all tissue types examined (the whole volume of interest, normal-appearing white matter, and cortical gray matter) in 23 RIS patients compared to a control group. The extent of the NAA/Cr decrease was impressive, on the order of 10%,^[8]^ which is even higher than that observed in some MS studies.^[33]^ By contrast, we observed either marginal or no abnormalities in metabolite concentrations in cortical gray matter of RIS patients, which is in line with the results of a previous 1H-MRS study inside the gray matter of MS patients, in whom metabolic alterations were present only in patients with inflammatory activity.^[34]^ Interestingly, the mean NAA concentrations in those RIS subjects with high risk for conversion to MS was ∼3.5% less than that found in both healthy controls and RIS patients without other characteristics for conversion to MS. The magnitude of this NAA decrease, albeit not significant, is close with the mean reduction in NAA concentrations of normal-appearing white matter in MS^[33]^ and in CIS^[35]^ observed in previous 1H-MRS studies performed with LCModel. Therefore, we cannot exclude the possibility of a small amount of axonal damage in those RIS subjects with high risk of developing MS. Although this result is in contrast with that reported by Stromillo et al,^[8]^ in which NAA/Cr ratio was not different between RIS patients with or without presence of risk factors of developing MS, it is more in accordance with previous CIS studies, which reported significantly lower NAA concentrations only in those CIS patients who showed an early conversion to definite MS.^[35,36]^

When compared with the study by Stromillo et al,^[8]^ ours differs on several aspects, including the following: (i) the use of a higher-field 1H-MRS operating at 3 T and a single shorter echo time acquisition; (ii) the use of several metabolite measures (absolute concentrations, metabolite ratios, and metabolite concentrations accounted for partial volume effect) rather than metabolites as a ratio to Cr; (iii) the investigation of a single-voxel located in the mid-parietal gray matter instead of a more comprehensive analysis that included a volume of interest located in the central brain (corpus callosum) as well as in voxels located in perilesional areas, normal-appearing white matter, and normal-appearing gray matter; and (iv) our voxel reflects a balanced mixture of gray matter and white matter signals (65% and 20% respectively) in contrast to pure gray matter signal of the interhemispheric gray matter voxel. Although these methodological differences hamper significantly direct comparisons between both studies, such a great difference in findings raise the question of current RIS studies limitations, ranging from the small sample sizes (an average of 20 RIS patients) to the possibility that subjects fulfilling the RIS criteria actually constitute a highly heterogeneous group.

In the present study, we also investigated differences in global brain volumes between RIS and healthy controls groups. Consistent with previous studies,^[6–10]^ we demonstrated significant global brain and cortical atrophy in RIS patients, unrelated to the presence of markers for conversion to future MS. The positive correlation observed between overall white matter lesion volume and whole cortical volume agrees with emerging literature on that retrograde damage of the perikarya of neurons due to injury of their axons (axonal transection) in the white matter lesions contributes significantly to gray matter atrophy.^[37]^ Nevertheless, our study was not specifically designed to assess to what extent white matter lesions contribute to regional cortical atrophy in RIS patients.

The study should be interpreted within the context of several limitations. First, the sample size was relatively small. Given the low incidence and prevalence of the disease, the RIS literature generally comprises studies with small sample sizes.^[6–11]^ The proportion of RIS patients with high risk for conversion to MS (6/18) accurately mirrors the proportion of RIS patients who developed future MS within 5 years in longitudinal studies,^[13]^ which means that our RIS sample might be representative of the general population with RIS. Another shortcoming in our study regards our 1H-MRS design for assessing metabolite alterations. Given the volume of 1H-MRS voxel utilized in this study (10 cm^3^), it was not possible to select pure cortical gray matter voxel and, consequently, the metabolic indices inside the voxel do not accurately reflect a pure gray matter analysis. Nevertheless, the gray matter represents almost ∼65% of the tissue present in the voxel, which can be considered well representative of gray matter metabolism.^[34]^ Furthermore, we only investigated 1 single-voxel and thus the normal metabolite concentrations found in our RIS patients do not necessarily reflect normal metabolite levels throughout the gray matter. This concept is further reinforced by the fact that our RIS patients showed whole cortical atrophy, which has been suggested to be explained by neuronal and axonal pathology.^[38]^ In this context, the inclusion of more voxels in different brain regions would have provided more accurate information of the RIS gray matter metabolite changes. Finally, cerebrospinal fluid data were only available in 50% of RIS subjects.

In conclusion, the main contributions of this study can be summarized as follows: (i) brain and cortical volumes were significantly lower in RIS patients than in controls; and (ii) occult microstructural normal appearing brain damage was not present beyond T2 visible lesions. Overall, our results suggest that in RIS, global brain and cortical atrophy is not primarily driven by significant occult microstructural normal appearing brain damage. Longitudinal MRI studies are needed to better understand the pathological processes underlying this novel entity.
